# *In vitro*-Formulated Oligomers of Strep-Tagged Avian Influenza Haemagglutinin Produced in Plants Cause Neutralizing Immune Responses

**DOI:** 10.3389/fbioe.2018.00115

**Published:** 2018-08-20

**Authors:** Hoang Trong Phan, Ulrike Gresch, Udo Conrad

**Affiliations:** Leibniz Institute of Plant Genetics and Crop Plant Research (IPK) Seeland, Germany

**Keywords:** avian flu, H5 oligomers, H5-step-tag trimers, strep-tactin® XT, neutralizing immune responses

## Abstract

The worldwide emergence of the novel influenza A H5N1 and H5N8 has notably and directly impacted the poultry industry, resulting in the need for effective and cheap vaccination strategies to protect poultry worldwide. Subunit vaccines from plants can be produced for a low cost, and plant production systems are easily scaled up at low infrastructure cost. However, subunit vaccines generally induce low immunogenicity against influenza. To address this issue, we present a new and innovative method to generate highly immunogenic H5 oligomers. The method is based on specific and high-affinity interaction between engineered streptavidin (Strep-Tactin® XT) and the Strep-tag II peptide. H5-Strep-tag II-tagged trimers were produced *via* transient agroinfection in tobacco leaves and purified, and oligomers were formulated *in vitro* by adding purified homotetrameric Strep-Tactin® XT. Immunogenicity was tested by performing mouse immunizations. Haemagglutinin oligomers produced *in vitro* by combining Strep-Tactin® XT and Strep-tag II-fused haemagglutinin trimers from plants raised potentially neutralizing antibodies in mice. Vaccines based on actual H5N1 haemagglutinin can be produced by combining strep-tagged haemagglutinin trimers from plants and Strep-Tactin® XT.

## Introduction

Emerging and re-emerging infectious diseases are often caused by zoonotic pathogens (Woolhouse and Gowtage-Sequeria, 2005). Prominent examples are swine flu H1N1 (World Health Organization., 2009) and avian flu [avian flu H5N1 (World Health Organization, 2018)]. Avian influenza viruses are highly pathogenic, relatively easily spread by their avian hosts, and capable of directly infecting humans. Therefore, they are candidates for the next global pandemic threat (Yen and Webster, 2009). A number of outbreaks of H5N1 and H5N8 in Southeast Asia and many European countries as well as the US and Canada have notably and directly impacted the poultry industry (FAO, 2005; European Centre for Disease Prevention Control, 2016), underlining the need for effective and cheap vaccination strategies to protect poultry worldwide. Such vaccines should be characterized by high efficacy and easily produced. Subunit vaccines from plants can be produced for a low price, and plant production systems are easily scaled up at low infrastructure cost (Topp et al., 2016). Agroinfiltration in tobacco species such as *Nicotiana benthamiana* has generally been demonstrated to be a suitable method for producing pharmaceutical proteins in plants (for review see Chen et al., 2013). We recently produced stable trimeric H5 haemagglutinins in the endoplasmic reticulum of tobacco leaf cells (Phan et al., 2013) by using an artificially designed trimerization domain (Harbury et al., 1993). The purified trimers induced neutralizing humoral immune responses in mice as shown by haemagglutination inhibition assays (Phan et al., 2013). In a follow-up paper, we described the production of H5 oligomers in plants. Oligomerization in the Endoplasmic Reticulum (ER) of plant cells was supported by the co-expression of trimeric H5 with S-Tag and S-protein with the TP element from IgM (Müller et al., 2013) in *N. benthamiana* leaves. We showed that specific neutralizing humoural immune responses were induced by immunization with leaf crude extracts in mice. Furthermore, H5 oligomers induced greater immunogenicity than trimers in terms of neutralizing antibody levels (Phan et al., 2017).

In this paper, we sought to determine how to produce oligomeric vaccines *in vitro* using a more specific and defined approach. We exploited the high-affinity interaction between engineered streptavidin (termed as Strep-Tactin® XT) and the Strep-tag II peptide (Voss and Skerra, 1997; Korndörfer and Skerra, 2002; IBA, https://www.iba-lifesciences.com/strep-tactin-xt-system-technology.html). Strep-Tactin® XT, the newly developed variant of Strep-Tactin® (Voss and Skerra, 1997; Korndörfer and Skerra, 2002), has a binding affinity in the nM range for Strep-tag II (IBA, https://www.iba-lifesciences.com/strep-tactin-xt-system-technology.htmlhref). This protein is a homotetrameric protein and commercially available in a purified form (IBA, https://www.iba-lifesciences.com/strep-tactin-xt-system-technology.htmlhref). Like Strep-Tactin®, each of the four subunits of Strep-Tactin® XT possesses a specific binding site for biotin as well as for Strep-tag II. Tetrameric Strep-Tactin® XT proteins serve as docking molecules to bind different Strep-tag II-fused protein molecules to form oligomers.

**Figure 1:**
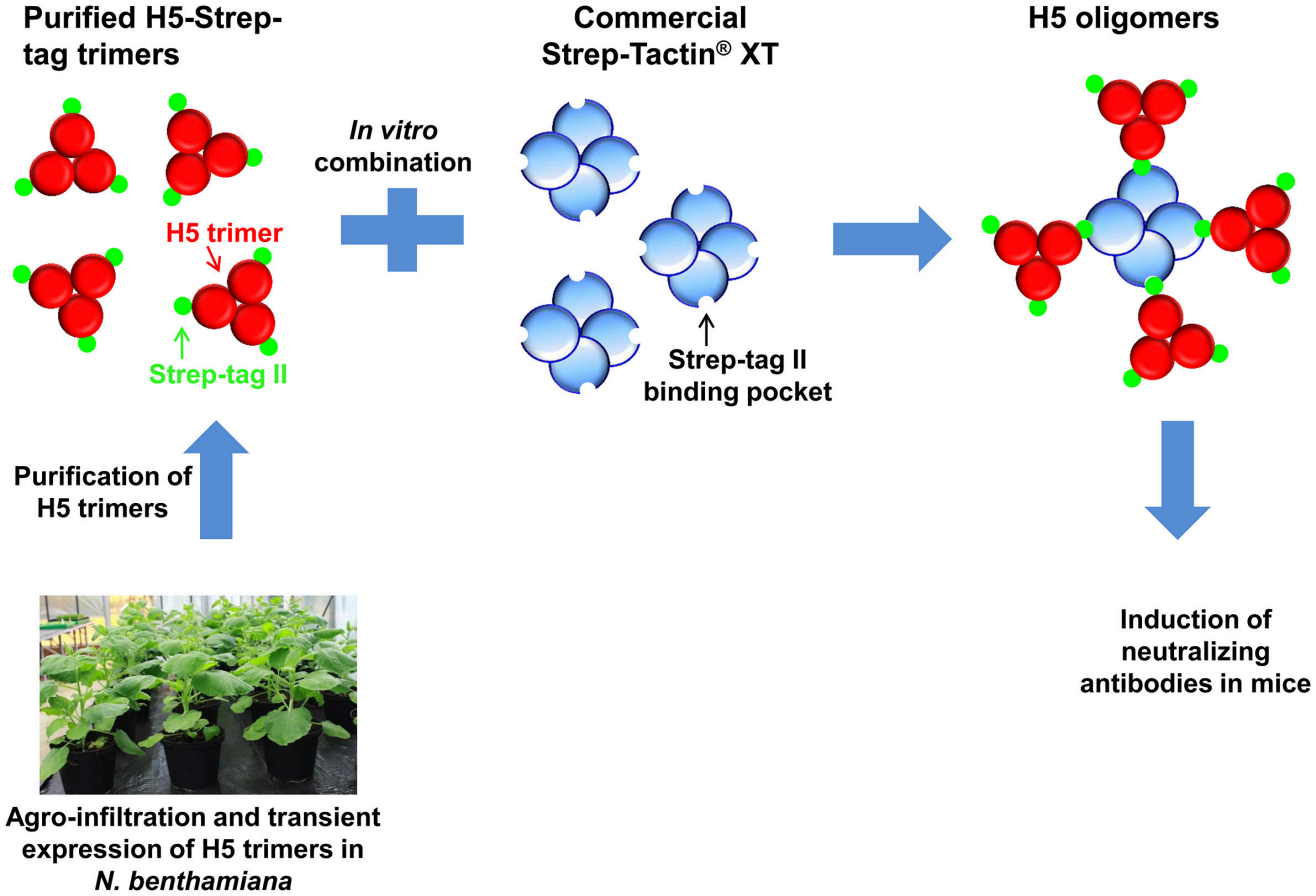
Model of H5 oligomer formation *via* the interaction between H5-Strep-tag trimers and tetrameric Strep-Tactin® XT. Haemagglutinin-Strep-tag II trimers are produced *in planta* and mixed *in vitro* with pure high-affinity Strep-Tactin® XT to form H5 oligomers. Oligomeric products with greater complexity than the example shown here are feasible.

Influenza haemagglutinin is a surface protein. Its native form is a homotrimeric protein, therefore we planned to keep its structure during oligomerization. To generate haemagglutinin oligomers *in vitro*, influenza haemagglutinin (H5) was fused with Strep-tag II sequence and trimerized by trimeric motif (Harbury et al., 1993). Haemagglutinin oligomers were formed by combining Strep-Tactin®XT and purified Strep-tag II-fused haemagglutinin trimers from plants (Figure 1). By this method, only the H5 component has to be newly prepared *in planta* if a new influenza viral strain appears. Here, we showed that H5 oligomers more effectively inducing neutralizing antibodies in mice than H5-Strep-tag trimers.

## Materials and methods

### Construction of plant expression vectors

**Figure 2:**
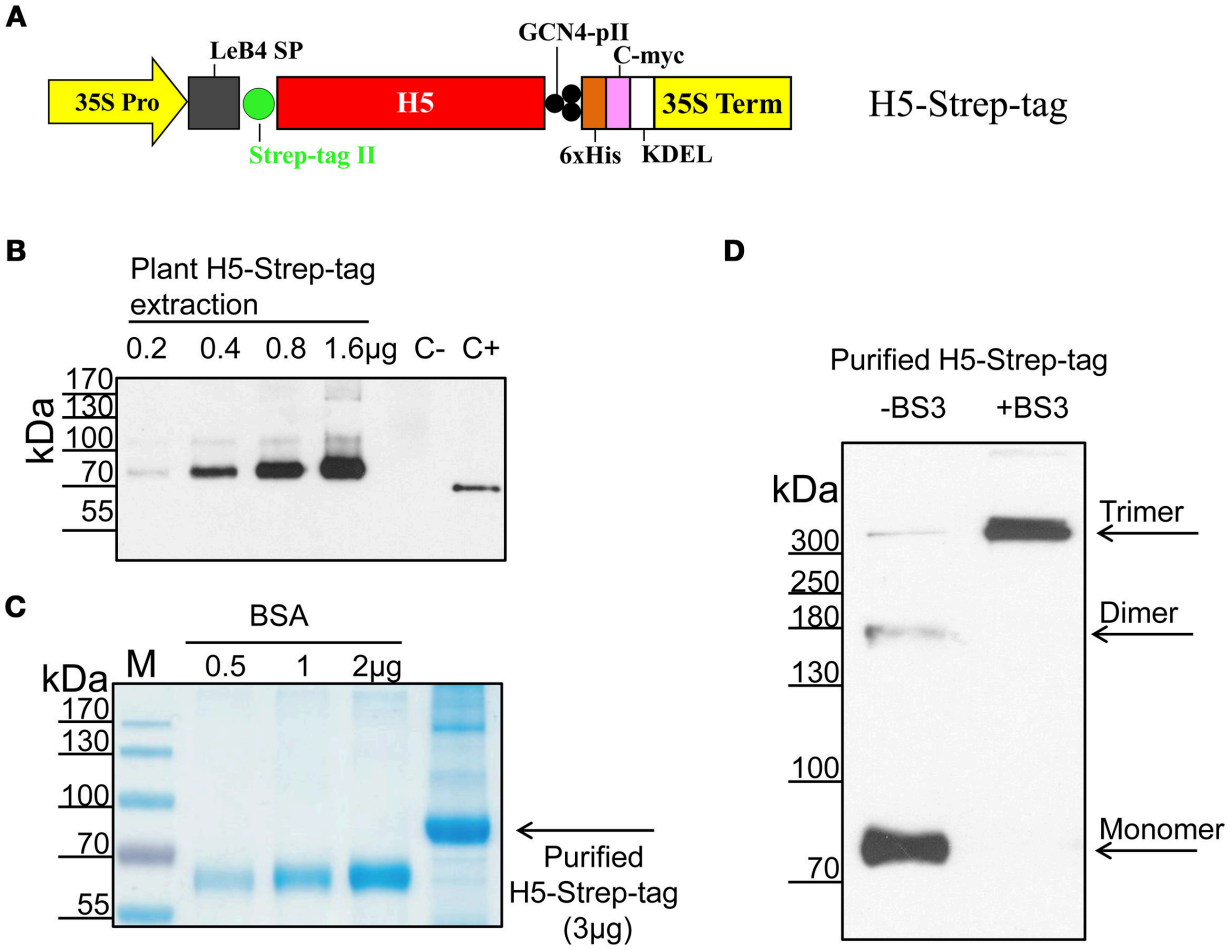
Expression, purification, and characterization of H5-Strep-tag from plants. **(A)** Expression casette for the production of H5-Strep-tag *in planta*. The ectodomain of haemagglutinin (H5) was C-terminally fused to a GCN4pII motif for trimerization, a c-myc tag for downstream detection by Western blot and a His tag for purification by IMAC. A Strep-tag sequence was N-terminally fused to the recombinant protein to facilitate oligomeric formation *via* interaction with Step-Tactin® XT. The legumin B4 signal peptide and the KDEL motif were used to promote the retention of transgene products in the endoplasmic reticulum. 35S Pro: Cauliflower mosaic virus 35S ubiquitous promoter; 35S Term: Cauliflower mosaic virus 35S terminator. **(B)** Expression of H5-Strep-tag confirmed by Western blot. Plant proteins from infiltrated leaf materials were extracted, separated by reduced SDS-PAGE, and analyzed by Western blot to detect anti-c-myc tags. One nanogram of standard anti-TNFalpha-nanobody-ELP (C+) (Conrad et al., 2011) was included as a positive control for Western blotting. C−: negative control, extracts of *Nicotiana benthamiana* plants infiltrated with an *Agrobacterial* strain harboring empty pCB-301 vectors. **(C)** H5-Strep-tag purification. Recombinant proteins were purified by IMAC. The concentration of the purified H5-Strep-tag was estimated by Bradford assay. Given amounts of recombinant protein and bovine serum albumin (BSA) were separated by reduced SDS-PAGE and visualized by Coomassie blue staining. **(D)** Oligomeric state of H5-Strep-tag based on a crosslinking reaction. Purified H5-Strep-tag trimer proteins were supplemented with 5 mM BS3 crosslinker (+BS3) or left untreated (-BS3) and were visualized by Western blotting with an anti-c-myc monoclonal antibody. Crosslinked products were assumed to be stable on SDS-PAGE gel under reducing, denaturing conditions.

The DNA sequence corresponding to amino acids 2–564 of the haemagglutinin of the A/duck/Viet Nam/TG24-01/2005 (H5N1) strain was synthesized commercially (GENECUST EUROPE, Luxembourg). Strep-tag II (WSHPQFEK) was added to the N-terminus of the aa17-520 H5 sequence. The resulting sequence was cloned into pRTRA-35S-H5pII (Phan et al., 2013) *via* BamHI and Bsp120I sites to form a recombinant vector designated pRTRA-H5-Strep-tag. This vector contained DNA sequences encoding the legumin B4 signal peptide, Strep-tag II, the haemagglutinin ectodomain, the trimeric GCN4-pII motif, a His tag, a c-myc tag, and the ER retention signal (KDEL). Expression of the H5 fusion protein was controlled by the CaMV 35S promoter and terminator. The expressed product was designated H5-Strep-tag (Figure 2A). The expression cassette of this vector was cloned into the pCB301 shuttle vector (Xiang et al., 1999) using HindIII restriction sites. pCB301 shuttle vectors were introduced into the *Agrobacterium* pGV2260 strain by electroporation.

### Agro-infiltration

Agro-infiltration for the expression of recombinant proteins was described in detail by Phan and Conrad (2016) and is briefly described here. *Agrobacteria* harboring shuttle vectors to express recombinant proteins (Figure 2A) and *Agrobacteria* harboring a plant vector to express HcPro, a suppressor of gene silencing (Sudarshana et al., 2006), were pre-cultivated separately in LB medium with 50 μg/mL kanamycin, 50 μg/mL carbenicillin, and 50 μg/mL rifampicin overnight at 28°C and 140 rpm. The pre-cultures were added to new LB medium containing the appropriate antibiotics. After cultivation for 24 h, bacteria were harvested by centrifugation (4,000 *g*, 30 min, 4°C) and resuspended in infiltration buffer [10 mM of 2-(N-morpholino) ethanesulfonic acid (MES), 10 mM of MgSO_4_, pH 5.6]. *Agrobacteria* harboring the shuttle vector for recombinant protein expression and the plant vector to express HcPro were combined and diluted in infiltration buffer to a final OD600 of 1.0. *N. benthamiana* plants (6 to 8 weeks old) were infiltrated by completely submerging each plant in an agrobacterium-containing cup standing inside a desiccator. A vacuum was applied for 2 min and then quickly released. The plants were then grown in the greenhouse at 21°C under 16 h of light per day. Five days after infiltration, leaf samples were harvested and stored at −80°C.

### SDS-page and western blotting

Extracted plant proteins, purified proteins, or an anti-TNFα-nanobody-ELP standard protein (Conrad et al., 2011) were separated by reducing SDS-PAGE (10% polyacrylamide) and then electrotransferred to nitrocellulose membranes. The Western blotting procedure was carried out using monoclonal anti-c-myc antibodies following the protocol described by Gahrtz and Conrad (2009). Horseradish peroxidase-conjugated sheep anti-mouse whole IgG was used as the secondary antibody (GE Healthcare UK limited, Little Chalfont Buckinghamshire, UK) followed by enhanced chemiluminescence-based (ECL) detection.

### Protein purification by IMAC

Five days after vacuum infiltration, leaf samples were harvested, frozen in liquid nitrogen and homogenized using a commercial blender. Total proteins from 80 g of infiltrated leaves were extracted in 240 ml of 50 mM Tris buffer (pH 8.0). The extracts were clarified by centrifugation (75,600 g, 30 min, 4°C) and then filtered through paper filters. The clarified extracts were mixed with 20 ml of packed Ni-NTA agarose resin that had previously been washed twice with water. After mixing for 30 min at 4°C, the mixture was added to a chromatography column. Thereafter, the column was extensively washed (50 mM of NaH_2_PO_4_, 300 mM of NaCl, 30 mM of imidazole, pH 8.0). Recombinant proteins were eluted from the column with elution buffer (50 mM of NaH_2_PO_4_, 300 mM of NaCl, 125 mM of imidazole, pH 8.0), placed in dialysis bags, concentrated in PEG 6 000 and dialyzed against PBS.

### Cross-linking of plant-derived H5-strep-tag

A cross-linking reaction was performed to determine the multimeric state of the plant-derived H5-Strep-tag proteins following the method described by Weldon et al. (2010). Briefly, 1 μg of purified plant-derived H5-Strep-tag proteins was mixed with Bis[sulfosuccinimidyl] suberate (BS3) to a 5 mM final concentration and incubated for 30 min at room temperature. The cross-linking reaction was stopped by the addition of 1 M Tris-HCl pH 8.0 to a final concentration of 50 mM and incubated for 15 min at room temperature. After cross-linking, proteins were separated on a 10% SDS-PAGE under reducing conditions, blotted and analyzed by Western blot using anti-c-myc monoclonal antibody.

### Formation of H5 oligomers *in vitro*

Strep-Tactin® XT (IBA Lifesciences, Goettingen, Germany) was mixed with H5-Strep-tag, resulting in final concentrations of 0, 1.6, 16, 160, 640 nM, 1.6, and 16 μM for Strep-Tactin® XT and 312.5 nm for H5-Strep-tag. Mixtures were rotated at room temperature for 30 min and used for the haemagglutination assay.

Oligomers formed by Strep-Tactin® XT and H5-Strep-tag at final concentrations of 160 nM and 312.5 nM, respectively, were designated H5 oligomers. These H5 oligomers were kept at 4°C to monitor their stability at different time points (1, 2, 6, and 36 days) by haemagglutination assay and to use for mouse immunization.

### Size exclusion chromatography

Purified H5-Strep-tag trimers or H5 oligomers at a final concentration of 63 μg/mL, containing 8.5 μg of Strep-Tactin® XT (the amount of Strep-Tactin®XT required to form 63 μg of H5 oligomers), were loaded onto a Superose™ 6 increase 10/300GL column (GE Healthcare). We used a high-molecular-weight kit containing standard proteins with molecular weights in the range of 44 kDa to 2,000 kDa, which were loaded onto the column to estimate the molecular weights of the proteins of interest. Five hundred microliters per fraction were collected for the haemagglutination test and Western blot analysis.

Plant-derived haemagglutinin trimers purified by IMAC were further purified by size exclusion chromomatography using a SuperoseTM 6 increase 10/300GL column (GE Healthcare Bio-Sciences AB Björkgatan 30 751 84 Uppsala, Sweden) to eliminate host plant proteins. The purity of plant-derived haemagglutinin trimers was analyzed by SDS-PAGE and visualized by Coomassie blue staining (Supplementary Material). Purified haemagglutinin trimers were used as an antigen source to coat ELISA plates.

### Mouse immunization

Twelve mice per group (9- to 10-week-old male C57/Bl6/J mice, Charles River Laboratories, Research Models and Services, Germany GmbH) were subcutaneously immunized with Emulsigen®-D adjuvant-formulated antigens [H5-Strep-tag (trimer)], Strep-Tactin® XT, H5 oligomer, and PBS buffer) on days 0, 14, and 28. One week after the 2nd and 3rd immunizations, mice were bled *via* the retro-orbital sinus. Mouse sera were collected individually for haemagglutination inhibition and ELISA tests. Ten micrograms of H5 content from either H5-Strep-tag trimers or H5 oligomers was used to immunize one mouse. In the control groups, twelve mice per group were immunized with 1.36 μg of Strep-Tactin®XT (the same amount of Strep-Tactin®XT was used to form 10 μg of H5 oligomers) or PBS buffer as negative control.

Antigens for one mouse were prepared in 160 μl volume and formulated with 40 μl of the Emulsigen®-D adjuvant (MVP Technologies, 4805 G Street, Omaha, NE 68117, USA) to give a final adjuvant concentration of 20%.

### Haemagglutination test and haemagglutination inhibition assay

The haemagglutination test was based on a standard protocol (World Organization for Animal Health, 2004). The dilution that induced complete haemagglutination was defined as one haemagglutination unit (HAU). The HI assay was performed similarly based on a standard procedure (World Organization for Animal Health, 2004). Due to the unavailability of the A/duck/Viet Nam/TG24-01/2005 (H5N1) virus in an inactivated form, the heterologous inactivated virus strain rg A/swan/Germany/R65/2006(H5N1) was used for the HI assay. The deduced haemagglutinin amino acid sequences are very similar in both strains (96%). A 25-μL aliquot of serum from a single mouse was placed in the first well of a microtiter plate containing 25 μL of PBS, and two-fold serial dilutions were made across the row of 8 wells. A 25-μL volume containing 4 HAU of the inactivated rg A/swan/Germany/R65/2006(H5N1) virus was added to the reaction and incubated at 25°C for 30 min. Then, 25 μL of 1% chicken red blood cells was added, and the plates were incubated at 25°C for 30 min. The HI titre is presented as the reciprocal of the highest dilution of serum that completely inhibited haemagglutination.

### Indirect ELISA

Microtitre plates (ImmunoPlateMaxisorp, Nalgen Nunc International, Roskilde, Denmark) were coated with 100 μL of 0.5 μg/mL IMAC- and SEC-purified haemagglutinin trimers diluted in Phage PBS (100 mM of NaCl, 32 mM of Na_2_HPO_4_, 17 mM of Na_2_HPO_4_, pH 7.2) and incubated overnight at room temperature. After blocking with 3% (w/v) bovine serum albumin (BSA) and 0.05% (v/v) Tween20 in PBS (PBST) for 2 h, 100 μL of a specific dilution (6 × 10^−4^) of serum was applied and incubated at 25°C for 1 h. Plates were washed 5 times with PBST, incubated with alkaline phosphatase-conjugated rabbit anti-mouse IgG diluted (diluted 2,000 times) in 1% (w/v) BSA and washed again. The enzymatic substrate, p-nitrophenyl phosphate (pNPP) in 0.1 M diethanolamine-HCl (pH 9.8) was added, and the absorbance signal was measured at 405 nm after incubation for 1 h at 37°C. Serum at a 6 × 10^−4^ dilution, which gave ELISA OD values of approximately 1, was included in every ELISA plate as an internal control and used to normalize ELISA values.

### Statistical analyses

Statistical analyses of HI data were performed using the Mann-Whitney Rank-Sum test in the Sigma Plot software. *P*-values < 0.05 were designated significant.

## Results

### Plant-based expression and purification of influenza haemagglutinin trimers

The H5 sequence encoding the ectodomain of H5 from the A/duck/Viet Nam/TG24-01/2005 (H5N1) strain was cloned into plant expression vectors under the control of the Cauliflower mosaic virus 35S ubiquitous promoter (CaMV 35S Promoter) and its terminator. A His tag (6 x histidine residues) and c-myc tag were C-terminally fused to the recombinant H5 protein to facilitate recombinant protein purification of expressed proteins by immobilized metal ion chromatography (IMAC) and detection by Western blot analyses, respectively. The legumin B4 signal peptide at the N-terminus and an endoplasmic reticulum (ER) retention signal (KDEL) at the C-terminus of the H5 sequence permitted the accumulation of recombinant proteins in the ER of leaf cells. The recombinant H5 proteins were fused to the Strep-tag II sequence and to a trimerization domain (GNC4-pII motif) at the N- and C-termini, respectively (Figure 2A). The authenticity of the expression vector was verified by sequencing.

This vector encoded a protein designated H5-Strep-tag. H5-Strep-tag proteins were expressed in *N. benthamiana* plants in transient expression experiments. Five days after vacuum infiltration, accumulation of the target proteins was confirmed by Western blot analyses, as demonstrated in Figure 2B. One specific band corresponding to approximately 80 kDa was visualized at different concentrations of H5-Strep-tag containing plant crude extracts after separation on a SDS gel under reducing conditions, while no Western blot signal was detected in plant extracts from plants infiltrated with *Agrobacteria* harboring empty plant shuttle vectors (Figure 2B). The sizes of the Western blot bands were higher than the expected molecular weight of the H5-Strep-tag monomer (66 kDa). This difference is probably due to N-glycosylation. As shown in previous studies, influenza H5 proteins contain N-linked oligosaccharides. These are bound to six potential N-linked glycosylation sites on H5 (Asn10, 11, 23, 154, 165, and 286) (Zhang et al., 2012; Phan et al., 2014).

H5-Strep-tag proteins were purified by IMAC and visualized on Coomassie blue-stained SDS gels run under reducing, denaturing conditions as a major band with an apparent molecular weight of 80 kDa and a minor band with a molecular weight of 170 kDa. These bands corresponded to a monomer and a dimer of haemagglutinin, respectively. The minor dimer band could be explained by incomplete denaturation (Figure 2C). Western blot analysis of purified H5-Strep-tag revealed that most H5-Strep-tag proteins were present in the form of an approximately 80-kDa band. Faint bands were detected at 170 kDa and approximately 300 kDa after separation under reducing, denaturing conditions; these sizes corresponded to monomers, dimers, and trimers of H5-Strep-tag, respectively (Figure 2D, -BS3 lane).

### Structural characterization of plant-derived H5-strep-tag

To determine the native form of purified H5-Strep-tag proteins, a crosslinking reaction was performed using a BS3 crosslinker. BS3 is a water-soluble and homo-bifunctional cross-linker which reacts with primary amines of target proteins to form stable amide bonds. When oligomeric proteins are exposed to BS3, amide bonds-crosslinks between each subunit of a multimeric protein are formed. This provides direct evidence for their close proximity. H5-Strep-tag proteins were exposed to BS3 and crosslinked products were separated on a SDS gel under reducing and denaturing conditions, blotted and immunodetected using anti-c-myc monoclonal antibody. Immunoblot results revealed a single band with an apparent molecular weight of approximately 300 kDa corresponding to a trimer (Figure 2D, +BS3 lane). This result implies, that the native structure of plant-derived H5-Strep-tag proteins is a H5-Strep-tag trimer.

Taken together, these data demonstrate that influenza haemagglutinin proteins containing the Strep-tag II sequence (H5-Strep-tag) were expressed and purified from infiltrated leaves as trimeric proteins resembling the native structure of influenza haemagglutinin. The trimeric H5-Strep-tag is essential for oligomer formation as shown in Figure 1.

### Formation of H5 oligomers *in vitro via* the interaction between strep-tag II and strep-tactin® XT

In this study, the specific high-affinity interaction between Strep-tag II and Strep-Tactin® XT was exploited to multimerize influenza H5-Strep-tag trimers. When tetrameric Strep-Tactin® XT proteins were introduced into trimeric H5-Strep-tag protein solution, specific binding between Strep-tag II from the H5-Strep-tag proteins and homotetrameric Strep-Tactin® XT is predicted to result in the formation of oligomers, as presented in Figure 1. To monitor the formation of haemagglutin oligomers, haemagglutination assay was employed as it is less time consuming and highly sensitive. The assay is based on the ability of influenza haemagglutinin to bind to sialic acid receptors presented on the surface of chicken red blood cells. Cross-linkages between influenza haemagglutinin and red blood cells cause the formation of a lattice called haemagglutination. Among haemagglutinin oligomers (Figure 1), cross-linkages between H5-Strep-tag trimers and Strep-Tactin® XT proteins were already created *via* the interaction between Strep-tag II and Strep-Tactin® XT, while influenza H5-Strep-tag trimers lacked these linkages. Logically, when haemagglutinin oligomers are mixed with a given amount of red blood cells, haemagglutination titres should be higher than those obtained with trimeric haemagglutinin.

In this study, the ratio of Strep-Tactin® XT to H5-Strep-tag was varied to achieve optimal oligomer formation. Different Strep-Tactin® XT concentrations (0, 1.6, 16, 160, 640, 1.6, and 16 μM) were combined with 312.5 nM of H5-Strep-tag at which haemagglutination started to be visible and this concentration of H5-Strep-tag corresponded to 10 μg of H5 antigens prepared in 160 μl. This preparation made the vaccine formulation with the Emulsigen®-D adjuvant and mouse vaccination more convenient to use. Ten micrograms of H5 antigen was previously shown to induce neutralizing antibodies in mice (Phan et al., 2013).

**Figure 3:**
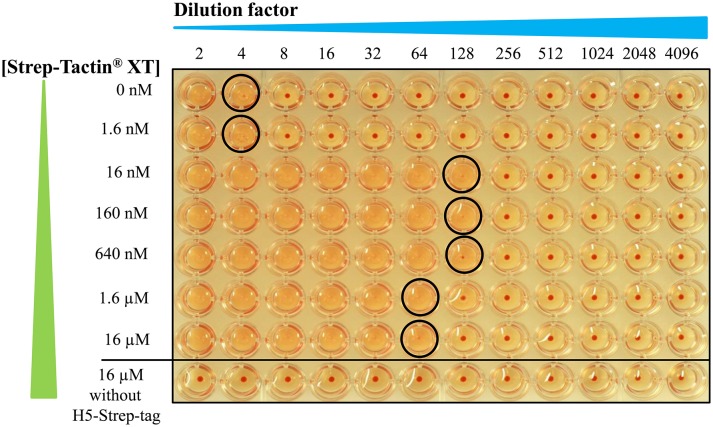
Formation of H5 oligomers based on the interaction of H5-Strep-tag trimers and Strep-Tactin® XT in a haemagglutination assay. H5-Strep-tag trimers were mixed with Strep-Tactin® XT at different concentrations (Strep-Tactin® XT from 0 to 16 μM and H5-Strep-tag trimers at 312.5 nM). Mixtures were incubated at room temperature for 30 min and then used for a haemagglutination assay. The circles indicate haemagglutination titres.

The different combinations of Strep-Tactin® XT with H5-Strep-tag were tested by a haemagglutination assay. As shown in Figure 3, haemagglutination titres of H5-Strep-tag alone and a mixture of H5-Strep-tag and 1.6 nM Strep-Tactin® XT were as low as 4, while Strep-Tactin® XT alone did not cause haemagglutination. The highest haemagglutination titres were observed when Strep-Tactin® XT concentrations between 1.6 and 640 nM were combined with the given concentration of H5-Strep-tag trimers. The slight reduction achieved by combining 1.6 μM Strep-Tactin® XT with 312.5 nM H5-Strep-tag was explained by the formation of very large oligomers causing protein precipitation. The oligomers produced by the combination of H5-Strep-tag trimers and Strep-Tactin® XT at 312.5 and 160 nM, respectively, were selected for further experiments (stability, size exclusion chromatography, and mouse immunization) and designated H5 oligomers. H5 oligomers were then kept at 4°C to test their stability. At different time points (30 min, 1, 2, 6, and 36 days), H5 oligomers were aliquoted for haemagglutination testing using haemagglutination assay. H5 oligomers retained their haemagglutnation activity as high as 128 until day 36. Oligomer formation between Strep-tag II-fused H5 trimers and Strep-Tactin® XT was stable over 1 month at 4°C.

Higher haemagglutination titres of H5-Strep-tag/Strep-Tactin® XT mixtures in comparison to those of H5-Strep-tag trimers could be explained by the difference in oligomeric states between them. To monitor different oligomeric states, purified H5-Strep-tag and H5 oligomers were further analyzed under native conditions thus keeping their oligomeric structure intact. Purified H5-Strep-tag, H5 oligomers, and Strep-Tactin® XT were separately analyzed by size exclusion chromatography (SEC) using a Superose™ 6 increase 10/300GL column. Proteins were sorted by their sizes and collected in different fractions.

**Figure 4:**
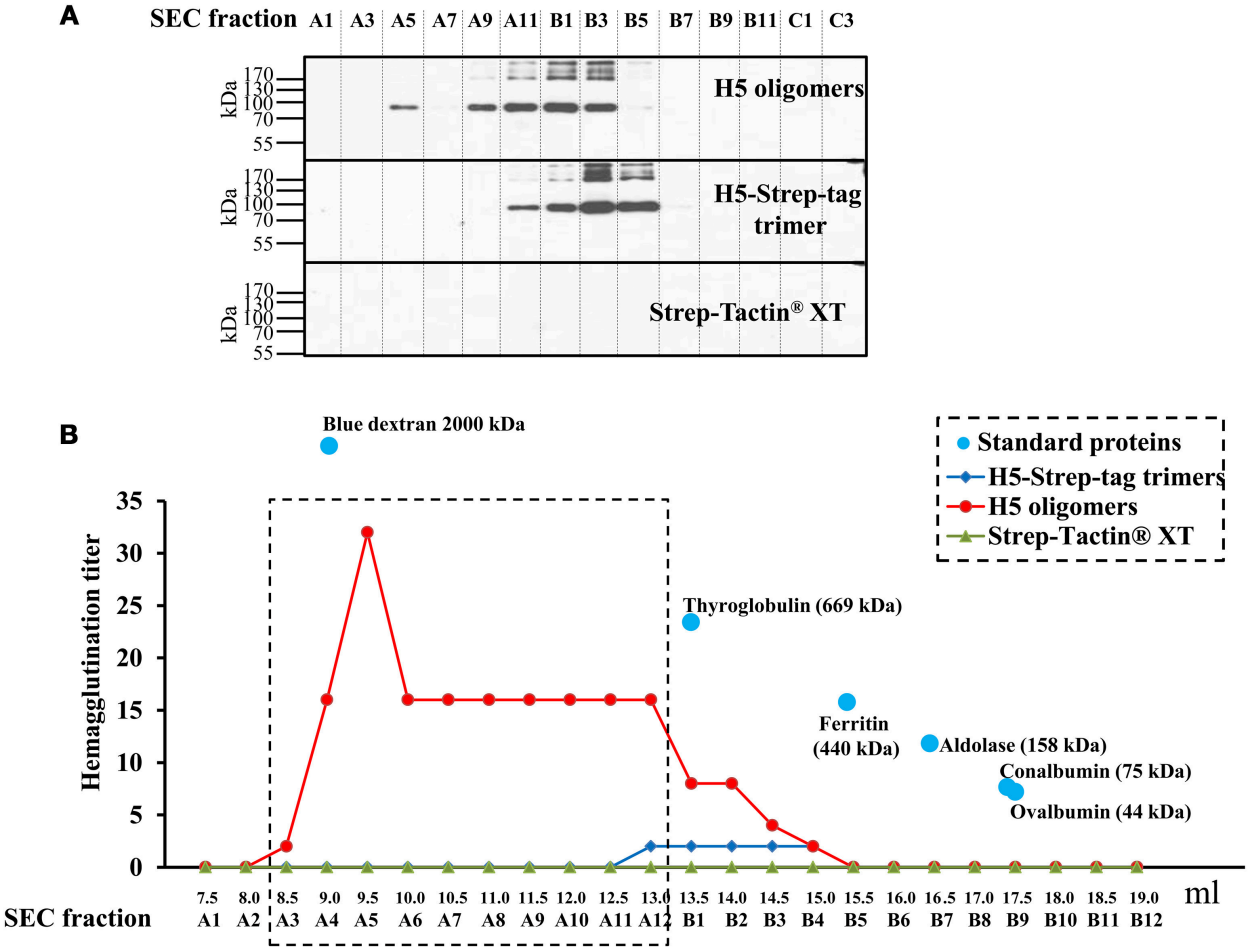
Characterization of H5 oligomers and H5-Strep-tag trimers by size exclusion chromatography. Starting amounts of each 63 μg in 0.5 ml of H5 oligomers, H5-Strep-tag trimers, and Strep-Tactin® XT were applied to a Superose™ 6 increase 10/300GL column. Haemagglutinin proteins were sorted by their sizes and collected in fractions. SEC fractions of H5 oligomers, H5-Strep-tag trimers, and Strep-Tactin® XT were analyzed by Western blot **(A)** and haemagglutination assay **(B)**. **(A)** Presence of haemagglutinin proteins in all SEC fractions was confirmed by Western blot analysis. Haemagglutinins in all SEC fractions of H5 oligomers, H5-Strep-tag trimers, and Strep-Tactin® XT were separated by SDS-PAGE under reducing, denaturing conditions and analyzed by Western blot using the anti-c-myc monoclonal antibody. Major bands with an apparent molecular weight of 80 kDa correspond to the size of monomers. Upper bands possibly represent dimers and larger sized multimers, not fully denaturated. **(B)** Distribution of haemagglutinins after SEC conducted by haemagglutination assay. The haemagglutination titre of each single fraction (from A1 to B12) of H5 oligomers, H5-Strep-tag trimers, and Strep-Tactin® XT was determined. The standard proteins loaded on the column to estimate the native molecular weights of target proteins are presented by light blue dots. Fractions from A3 to A12 showing different haemagglutination titres between H5 oligomers and H5-Strep-tag trimers were highlighted in the dash line rectangle.

Fractions A1 to C3 of each separation (H5 oligomers, H5-Strep-tag, and Strep-Tactin® XT, respectively) were analyzed by Western blot using an anti-c-myc monoclonal antibody under reducing, denaturing conditions. As presented in Figure 4A (the first panel), H5 oligomers were present in earlier A5 to B3 SEC fractions. This result indicated a very high molecular weight of H5 oligomers. H5-Strep-tag (Figure 4A, middle panel) was mainly distributed in fractions A11 to B5. The majority of trimeric proteins were distributed in fractions B3 to B5. As expected, a c-myc-tag containing protein was not present in all SEC fractions of Strep-Tactin® XT (Figure 4A, the last panel). The functionality of haemagglutinin in all fractions was determined by performing a haemagglutination assay.

As demonstrated in Figure 4B, early SEC fractions (A4 to A12) of the H5 oligomers showed the highest haemagglutination titres (max. 32). The sizes of the H5 oligomers in these fractions ranged from 669 to approximately 2 000 kDa. Haemagglutination titres were lower in fractions B1–B4 SEC fractions. From fraction B5 to B12 no haemagglutination was observed.

The analysis of H5-Strep-tag fractions showed only low haemagglutination titres from A12 to B4. In these fractions H5-Strep-tag trimers were detected (Figure 4). As expected, haemagglutination was negative in all SEC fractions of Strep-Tactin® XT (Figure 4B).

Both haemagglutination and Western blot results of SEC fractions indicated that H5 oligomers were really oligomeric haemagglutinins which showed high molecular weight, while H5-Strep-tag exclusively consisted of trimers. The H5 oligomer formation process was carried out by integrating different numbers of H5-Strep-tag trimers into the binding sites of Strep-Tactin® XT molecules.

### High immunogenicity of H5 oligomers

**Figure 5:**
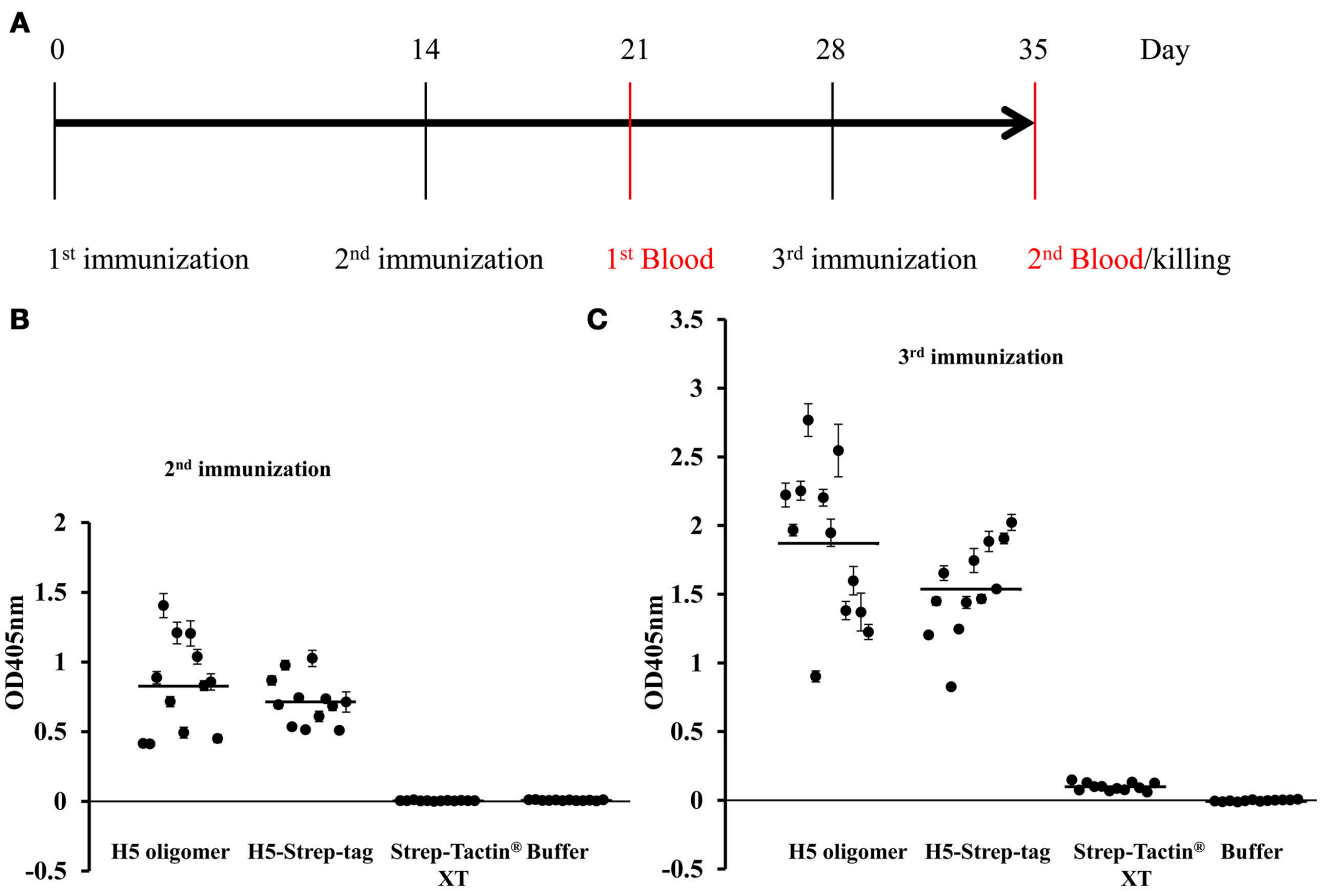
Mouse immunization experiments. **(A)** Mouse vaccination schedule. Twelve mice per group [9- to 10-week-old male BL6 (C57/Black6J)] were immunized subcutaneously with H5 oligomers, H5-Strep-tag trimers, Strep-Tactin® XT or buffer on days 0, 14, and 28. Each mouse in groups 1 and 2 received 10 μg of H5 content from either H5 oligomers or H5-Strep-tag trimers. Each mouse in group 3 was vaccinated with 1.36 μg of Strep-Tactin® XT; this amount corresponds to the amount of Strep-Tactin® XT in 10 μg of H5 oligomers. In the control group, mice were immunized with the PBS buffer used for the binding reaction between Strep-Tactin® XT and H5-Strep-tag trimers. All antigens and control buffers were formulated with Emulsigen®-D adjuvant. Mice were retro-orbitally bled 1 week after the 2nd and 3rd immunizations. Mouse sera were collected individually for haemagglutination inhibition (HI) and ELISA tests. **(B,C)** Mouse antibody responses determined by ELISA. Antibody responses after the 2nd **(B)** and 3rd immunizations **(C)**. Plates coated with 50 ng of purified haemagglutinin trimers per well were incubated with mouse sera at a 6 × 10^−4^ dilution. The plates were then incubated with alkaline phosphatase-conjugated rabbit anti-mouse IgG. Enzyme activity was determined using pNPP. The absorbance signal was measured at 405 nm. A single dot indicates the ELISA value for a single mouse. Bars indicate the average value of each test group.

The purpose of this experiment was to determine whether haemagglutinin trimers or haemagglutinin oligomers induce strong immune responses, particularly neutralizing antibodies. All mouse sera from four groups (twelve mice per group) were collected to detect target-specific antibody responses by ELISA and neutralizing antibodies by HI tests after the second and third immunizations (Figure 5A).

To measure the haemagglutinin-specific immune responses induced by H5-Strep-tag trimers and H5 oligomers by ELISA, haemagglutinin trimers (without the Strep-tag II sequence) (Phan et al., 2017) were purified by IMAC and further purified by size exclusion chromatography to remove plant proteins. High purity of purified plant-derived haemagglutinin trimers was confirmed by Coomassie blue staining (Supplementary Material). Purified haemagglutinin trimers at 50 ng/well were absorbed onto polystyrol ELISA plates. Then, equal dilutions (6 × 10^−4^) of individual mouse sera from all mice were applied. The addition of a secondary alkaline phosphatase-conjugated anti-mouse IgG antibody allowed the quantitative detection of target-specific mouse antibodies. As shown in Figure 5B, all mice immunized twice with 10 μg of H5 oligomers and H5-Strep-tag trimers developed H5-specific immune responses, while control mouse sera (from mice vaccinated with Strep-Tactin®XT and PBS) did not show any immune responses against H5 (Figure 5B). Immune responses were augmented by the third immunization (Figure 5C). Haemagglutinin H5 specific antibodies were observed after the third immunization with H5 oligomers and H5-Strep-tag, while no immune response against haemagglutinin H5 was, as expected, detected after immunization with Strep-Tactin®XT and PBS (Figure 5C).

**Figure 6:**
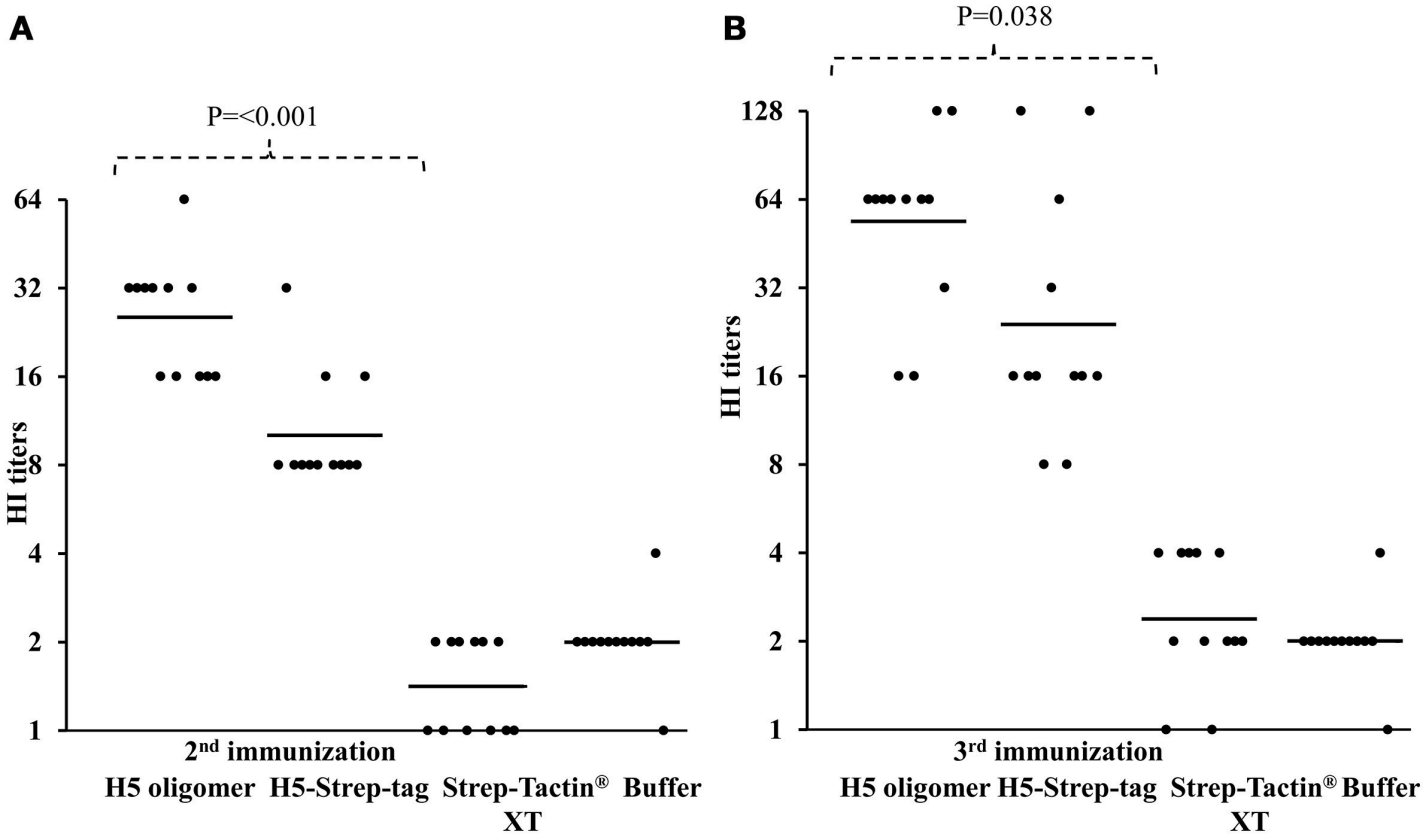
HI titres against rg A/swan/Germany/R65/2006(H5N1) in the sera of mice vaccinated two **(A)** or three times **(B)**. HI titres were expressed as the reciprocal of the highest dilution of serum that inhibited the haemagglutination of four haemagglutination units of rg A/swan/Germany/R65/2006(H5N1). Each black dot represents the HI titre of a single mouse serum sample. Bars indicate the geometric means of the titres of individual groups.

The activity of neutralizing antibodies induced by different forms of haemagglutinin was measured in an HI assay. All sera from the experimental group of mice vaccinated with 10 μg of H5-Strep-tag trimers after the second immunization demonstrated positive HI titres (≥ 8), and the geometric mean titre (GMT) of this group was 10.08. All sera from mice that received 10 μg of H5 oligomers showed HI titres higher than 16, and the GMT reached 25.40. The GMTs of mice vaccinated with Strep-Tactin® XT alone or buffer were 1.41 and 2.00, respectively. These results indicated that both the H5-Strep-tag trimers and H5 oligomer induced neutralizing antibodies against inactivated virus. However, mice vaccinated with H5 oligomers induced higher levels of neutralizing antibodies than those triggered by H5-Strep-tag trimers. This difference was significant (*P* = < 0.001; Figure 6A). After the third immunization, all sera from mice vaccinated with 10 μg of H5-Strep-tag trimers or H5 oligomers exhibited higher HI titres than those obtained after the second immunization. The GMTs of sera from mice vaccinated with H5 oligomers and H5-Strep-tag trimers rose to 53.82 and 23.98, respectively, while the GMTs of sera from mice vaccinated with Strep-Tactin® XT alone or buffer remained low. Notably, the difference between the titres obtained with H5-Strep-tag trimers and H5 oligomers was significant (*P* = 0.038; Figure 6B). Taken together, higher levels of neutralizing antibodies were induced by H5 oligomers compared to H5-Strep-tag trimers.

## Discussion and conclusion

Egg-based vaccines have been used to combat different seasonal influenza for many years, but the length of the production cycle and the limited manufacturing capacity available for this type of vaccine would not permit the rapid and efficient response necessary during a potential influenza virus pandemic (Wei et al., 2008). Cell culture technology as well as plant-based production of purified haemagglutinin trimers and oligomers have been shown to induce neutralizing immune responses in mice (Wei et al., 2008; Phan et al., 2013, 2017). In a new approach we present here, a commercially available protein (Strep-Tactin® XT) was used to form haemagglutinin oligomers from trimers using Strep-tag *in vitro*. Thus, only one haemagglutinin construct representing the version from a pathogenic influenza virus was transiently expressed in plants. Methods and systems for foreign protein production in plants have been extensively improved in the last 25 years (for reviews, see Wilken and Nikolov, 2012; Floss and Conrad, 2013). A transient expression system in *N. benthamiana* plants, an Australian wild tobacco species, was previously demonstrated to permit influenza vaccine production. Using this system, the first lot of purified research-grade proteins is available 22 days after initiating production [for a review, see (D'Aoust et al., 2010)]. Candidate vaccines produced with this system have been shown to be safe, effective in pre-clinical trials and easy to scale up for further production (D'Aoust et al., 2008). The virus production strategy proposed here permits the combination of strep-tagged seasonal haemagglutinin variants with Strep-Tactin® XT. This allows for the formation of oligomers which induce neutralizing antibody responses higher than those induced by trimers. The efficacy of this general strategy has to be tested for each single case. Here, especially in the case of applications to produce human influenza vaccines, the potential immunogenicity of Strep-Tag and Strep-Tactin® XT needs to be further evaluated in detail. The essential components of the basic technology used for plant-based oligomeric vaccine production do not require alteration. Furthermore, different haemagglutinin variants can be mixed, facilitating the combined production of multivalent oligomeric vaccines. The efficacy of such oligomeric mixed vaccines will be tested in future experiments.

## Ethics statement

The animal experiments were approved by the Landesverwaltungsamt Sachsen-Anhalt, Halle/Saale, Referat Verbraucherschutz, Veterinärangelegenheiten and by the Landkreis Harz, Amt für Veterinärwesen und Lebensmittelüberwachung, Halberstadt. All animals received humane care according to the requirements of the German Animal Welfare Act, §8 Abs. 1.

## Author contributions

HP and UC conceived the study and designed the experiments. UG and HP performed experiments. UC and HP wrote the manuscript.

### Conflict of interest statement

The authors declare that the research was conducted in the absence of any commercial or financial relationships that could be construed as a potential conflict of interest.
