# Supplementary material for: In vitro-Formulated Oligomers of Strep-Tagged Avian Influenza Haemagglutinin Produced in Plants Cause Neutralizing Immune Responses
Source: Front Bioeng Biotechnol. 2018 Aug 20;6:115. doi: 10.3389/fbioe.2018.00115 (PMC6110258; doi:10.3389/fbioe.2018.00115)
Supplement: Supplementary file 1 [file Image_1.pdf]

Supplementary Material

***In vitro*-formulated oligomers of strep-tagged avian influenza haemagglutinin produced in plants cause neutralizing immune responses**

Hoang Trong Phan, Ulrike Gresch and Udo Conrad

Leibniz Institute of Plant Genetics and Crop Plant research (IPK) Gatersleben, Corrensstrasse 3, D-06466 Seeland, OT Gatersleben, Germany

H.T.P: hoang@IPK-gatersleben.de; U.C: conradu@IPK-gatersleben.de (corresponding author)

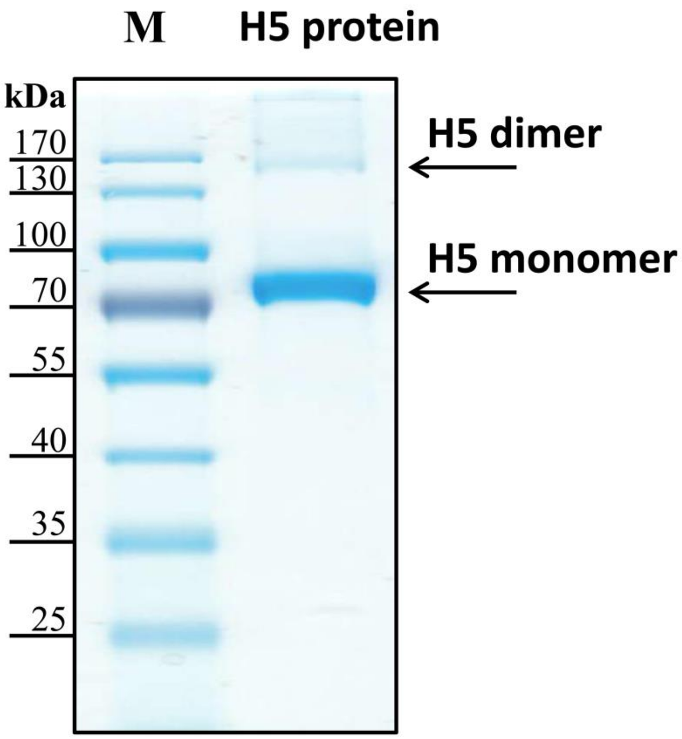

Supplementary Figure 1. Purity of plant-derived haemagglutinin trimers, as demonstrated by Coomassie blue stain. Plant-derived haemagglutinin trimers (Phan et al., 2017) were purified by IMAC, then further purified by size exclusion chromomatography using a SuperoseTM 6 increase 10/300GL column (GE Healthcare Bio-Sciences AB Björkgatan 30 751 84 Uppsala, Sweden) to eliminate host plant proteins. 2.5 µg of purified haemagglutinin trimers were then separated by SDS-PAGE (10% PAA) at reducing and denaturing condition, and visualized by Coomassie blue staining.

As shown in Supplementary Figure 1, haemagglutinin trimers (Phan et al., 2017) purified by both IMAC and size exclusion chromatography from infiltrated leaves were highly pure. The pure hemagglutinins were absorbed onto polystyrol ELISA plates to detect H5-specific mouse antibodies induced by H5-Strep tag and H5 oligomers.

References

Phan, H. T., Ho, T. T., Chu, H. H., et al. (2017). Neutralizing immune responses induced by oligomeric H5N1-hemagglutinins from plants. *Vet Res* 48, 53.
